# Co-option of bacteriophage lysozyme genes by bivalve genomes

**DOI:** 10.1098/rsob.160285

**Published:** 2017-01-18

**Authors:** Qian Ren, Chunyang Wang, Min Jin, Jiangfeng Lan, Ting Ye, Kaimin Hui, Jingmin Tan, Zheng Wang, Gerald J. Wyckoff, Wen Wang, Guan-Zhu Han

**Affiliations:** 1 Jiangsu Key Laboratory for Biodiversity and Biotechnology, Jiangsu Key Laboratory for Microbes and Functional Genomics, Jiangsu Engineering and Technology Research Center for Microbiology, College of Life Sciences, Nanjing Normal University, Nanjing 210046, People's Republic of China; 2 State Key Laboratory Breeding Base of Marine Genetic Resource, Third Institute of Oceanography, SOA, Xiamen 361005, People's Republic of China; 3 State Key Laboratory of Crop Biology, College of Agronomy, Shandong Agricultural University, Tai'an, Shandong 271018, People's Republic of China; 4 College of Fisheries, Huazhong Agricultural University, Wuhan, Hubei 430070, People's Republic of China; 5 College of Life Sciences, Zhejiang Sci-Tech University, Hangzhou 310018, People's Republic of China; 6 Divison of Molecular Biology and Biochemistry, School of Biological Sciences, University of Missouri-Kansas City, 5100 Rockhill Rd., Kansas City, MO 64110, USA

**Keywords:** bivalves, lysozymes, molecular evolution, bacteriophage

## Abstract

Eukaryotes have occasionally acquired genetic material through horizontal gene transfer (HGT). However, little is known about the evolutionary and functional significance of such acquisitions. Lysozymes are ubiquitous enzymes that degrade bacterial cell walls. Here, we provide evidence that two subclasses of bivalves (Heterodonta and Palaeoheterodonta) acquired a lysozyme gene via HGT, building on earlier findings. Phylogenetic analyses place the bivalve lysozyme genes within the clade of bacteriophage lysozyme genes, indicating that the bivalves acquired the phage-type lysozyme genes from bacteriophages, either directly or through intermediate hosts. These bivalve lysozyme genes underwent dramatic structural changes after their co-option, including intron gain and fusion with other genes. Moreover, evidence suggests that recurrent gene duplication occurred in the bivalve lysozyme genes. Finally, we show the co-opted lysozymes exhibit a capacity for antibacterial action, potentially augmenting the immune function of related bivalves. This represents an intriguing evolutionary strategy in the eukaryote–microbe arms race, in which the genetic materials of bacteriophages are co-opted by eukaryotes, and then used by eukaryotes to combat bacteria, using a shared weapon against a common enemy.

## 1. Introduction

It has been well established that horizontal gene transfer (HGT), the movement of genetic materials between distinct evolutionary lineages, plays an essential role in the evolution of prokaryotic genomes [1–3]. Moreover, during the establishment of the mitochondrion and the plastid, large amounts of genetic material from the endosymbionts were transferred to the eukaryotic nuclear genomes. In contrast, the ongoing, subsequent HGT events in eukaryotes have long been underappreciated [3]. Recently, more and more cases of HGT have been reported in eukaryotes [3].

Lysozymes are ubiquitous bacteriolytic enzymes found in most life forms and viruses, hydrolysing peptidoglycans in bacterial cell walls [4–6]. Several distinct lysozyme classes have been described, including hen egg-white lysozyme (HEWL; glycoside hydrolase 22 [GH22]), goose egg-white lysozyme (GEWL or GH23), bacteriophage T4 lysozyme (T4 L or GH24) and GH25 lysozyme typically found in bacteria [5,7–9]. Different classes of lysozymes lack any obvious similarity at the sequence level, but share certain (albeit distant) similarity in their three-dimensional structures [4]. Essentially degrading bacterial cell walls, lysozymes function in various biological processes, such as defence of bacterial infections (animals and plants), digestion of bacteria as food (animals and protozoa), cell wall synthesis and remodelling (bacteria), and lysis of bacteria at the end of the phage replication cycle [8].

Recently, GH25 lysozyme was found to be repeatedly transferred from bacteria to fungi, insects, plants and archaea [10]. The horizontally transferred lysozymes are used as antibacterial molecules by recipients and may thus complement the recipients' response to bacteria [10]. However, much remains unknown about both their evolutionary and functional implications. In a specific potential case of HGT, a phage-type-like lysozyme (GH24 lysozyme) was identified in the Manila clam (*Ruditapes philippinarum*) [9], but the evolutionary history and mechanism of acquisition of this lysozyme have not been well established. HGT was suggested to be ‘the most probable explanation’ [9]. However, the possibility of this lysozyme being a laboratory artefact or contaminant has not been formally excluded.

In this study, we provide evidence that phage-type GH24 lysozyme genes were horizontally transferred into bivalve genomes several hundred million years ago. The co-opted lysozyme genes have undergone dramatic gene structural changes and multiple independent gene duplication events. We also provide evidence that the co-opted phage-type lysozymes have an antibacterial function in their new hosts. This may be a general strategy for evolutionary co-option of specific weapons from the arsenal of one species using HGT, and understanding this case will help advance our understanding of the mechanisms and evolutionary pressures surrounding HGT in eukaryotes.

## 2. Results

### 2.1. Identification of phage lysozyme-like genes in the bivalve genomes

**Figure 1.**
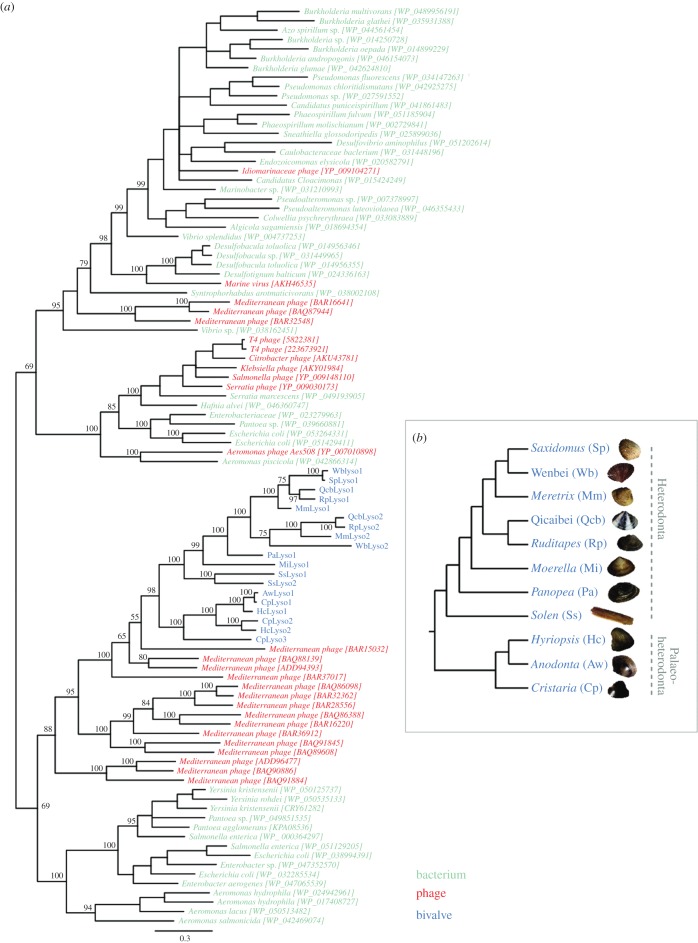
(*a*) The phylogenetic tree of bacterial, phage and bivalve lysozymes. The bacterial, phage and bivalve lysozymes are highlighted in turquoise, red and blue, respectively. The topology was inferred using a Bayesian methodology. The numbers on the selected nodes represent Bayesian posterior probabilities. (*b*) The bivalve phylogeny is based on the one inferred using the *cox1* gene (electronic supplementary material, figure S1). Bivalve abbreviations: Sp, *Saxidomus purpuratus*; Mm, *Meretrix meretrix*; Rp, *Ruditapes philippinarum*; Mi, *Moerella iridescens*; Pa, *Panopea abrupta*; Ss, *Solen strictus*; Wb, Wenbei; Qcb, Qicaibei; Hc, *Hyriopsis cumingii*; Cp, *Cristaria plicata*; Aw, *Anodonta woodiana*.

Through analysis of the transcriptome sequences of various bivalves and experimental work, we uncovered 19 sequences that share significant similarity with GH24 phage-type lysozyme in 11 bivalves (figure 1; electronic supplementary material, tables S1 and S2). The 11 bivalve species used in this study belong to two subclasses of Bivalvia: Heterodonta and Palaeoheterodonta (figure 1; electronic supplementary material, table S1). Because Qicaibei and Wenbei have not been classified and do not have Latin names yet, we used their Chinese pinyin names instead. Phylogenetic analysis shows that both Wenbei and Qicaibei belong to the subclass of Heterodonta (electronic supplementary material, figure S1). Similarity searches against the complete genomic sequence of *Crassostrea gigas* (subclass Pteriomorphia) yielded no significant hit to these bivalve lysozymes, suggesting that not all the bivalve species contain the GH24 phage-type lysozyme (discussed below). Ding *et al.* [9] identified one phage-type lysozyme sequence in *R. philippinarum*. We have identified two phage-type lysozyme sequences in *R. philippinarum*, namely RpLyso1 and RpLyso2. One of them, RpLyso1, shared approximately 98% nucleotide identity with Ding *et al.*'s sequence [9].

**Figure 2.**
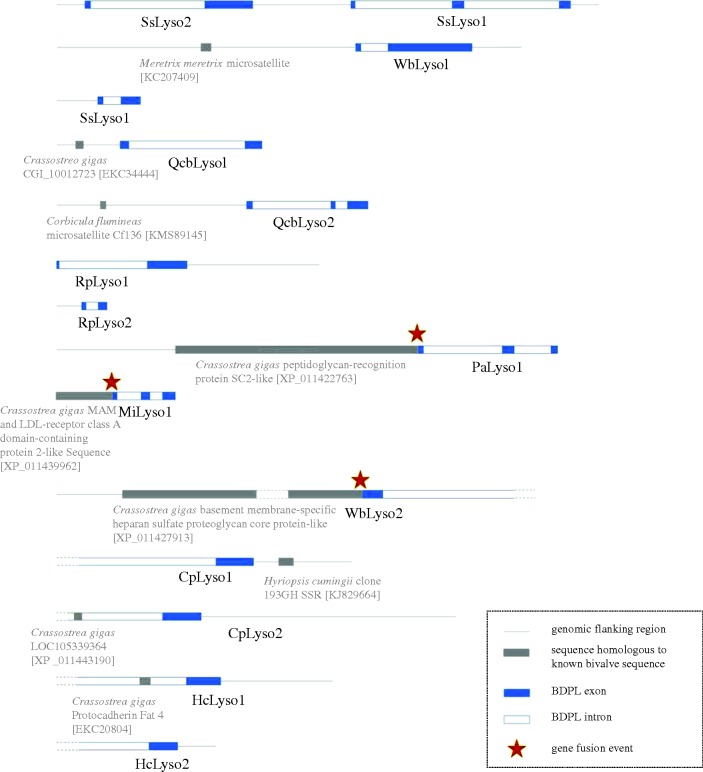
The flanking regions and gene structures of the *BCPL* genes. The white and blue rectangles represent the exons and introns of the *BCPL* genes, respectively. The grey rectangles represent the flanking regions that share significant similarity with known bivalve sequences. The dashed lines indicate incomplete regions. The blue dashed lines indicate incomplete introns, which were inferred based on the complete mRNA sequences. The best similarity search hits were labelled under the grey rectangles. Orange stars indicate the gene fusion events.

To rule out the possibility of laboratory contamination, we used the genome walking approach to isolate flanking genomic fragments adjacent to these bivalve lysozyme genes. We successfully sequenced the flanking regions of 15 lysozyme sequences. We found nine lysozyme sequences are flanked by sequences that share significant similarity with other known bivalve sequences, such as microsatellite sequences, *peptidoglycan-recognition protein* (*PGRP*) gene and *Protocadherin Fat 4* gene (figure 2). The lysozyme genes of *Solen strictus* (i.e. *SsLyso1* and *Sslyso2*) are tandem. Moreover, all the bivalve lysozyme genes identified here contain one or more introns (discussed below; figure 2). These multiple and independent lines of evidence suggest that the phage-type lysozyme-like sequences within the bivalve genomes are not artefacts or contaminants.

### 2.2. The origin of bivalve lysozyme genes

To investigate the origin of the phage T4 lysozyme-like sequences in the bivalve genomes, we conducted phylogenetic analyses of the lysozyme protein sequences of phages, bacteria and bivalves. We found that the bivalve lysozyme sequences form a monophyletic group with strong statistical support: Bayesian posterior probability (PP) = 0.98. The phylogenetic tree also shows that the bivalve lysozyme clade robustly nests within the clade of phage lysozymes (PP = 1.00). The closely related taxa are the phages isolated in the Mediterranean sea [11,12]. The phylogenetic pattern indicates that these bivalve lysozyme sequences ultimately originated from bacteriophages. We thus designate these bivalve sequences *bivalve co-opted phage lysozyme-like* (*BCPL*) genes.

### 2.3. Frequent gene structure changes after co-option

Phage lysozyme genes do not contain any introns. To our surprise, the *BCPL* genes contain at least one intron (figure 2). For *WbLyso2*, *CpLyso1/2*, *HcLyso1/2* genes, we were able to obtain only partial gene sequences, but inferred that they contain at least one intron based on mRNA sequence information (figure 2). This represents a compelling example of intron gain in eukaryotic species. However, we do not observe any general trend for the intron gain and the evolution of intron sizes. The introns do not share any significant similarity with each other, indicating they are not under strong functional constraint.

We also observed at least three potential gene fusion events involving the *BCPL* genes (figure 2; electronic supplementary material, figure S2). The *BCPL* genes (*PaLyso1*, *MiLyso1* and *WbLyso2*) were fused into novel genes with the *PGRP* gene, the *MAM and LDL-receptor class A domain-containing protein 2-like* (*MALRD2*) gene and the *basement membrane-specific heparan sulfate proteoglycan core protein-like* (*HSPG*) gene in *Panopea abrupta*, *Moerella iridescens* and Wenbei, respectively. All three fusion genes can be expressed as a single transcript. Interestingly, for the *PGRP-PaLyso1* fusion gene, the *Palyso1* can be expressed independently (electronic supplementary material, figure S2).

### 2.4. Recurrent gene duplication of bivalve lysozyme genes

Our phylogenetic analysis shows that at least four independent gene duplication events occurred within the *BCPL* genes: (i) one in the common ancestor of *Ruditapes philippinarum*, *Meretrix meretrix*, *Saxidomus purpuratus*, Qicaibei and Wenbei; (ii) two in the common ancestor of *Hyriopsis cumingii*, *Cristaria plicata* and *Anodonta woodiana*; and (iii) one in the lineage leading to *S. strictus*.

The absence of related lysozyme gene copies (figure 1), namely *SpLyso2*, *AwLyso2*, *AwLyso3* and *HcLyso3*, might be because of either gene loss or failure of transcriptome sequence due to the specific expression patterns of related genes. Either possibility does not change our conclusions regarding the recurrent nature of these duplication events.

### 2.5. Antibacterial activity of bivalve co-opted phage lysozyme-like genes

**Figure 3.**
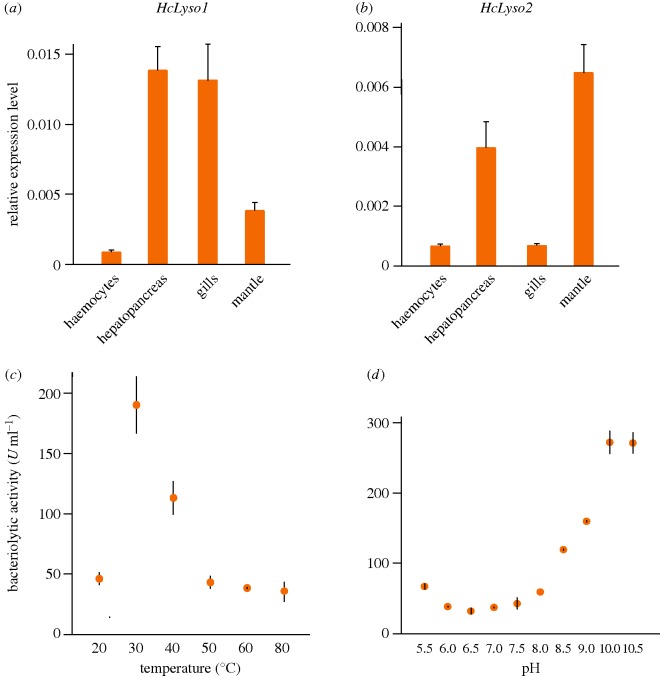
Expression and functionality of the *H. cumingii BCPL* genes. The gene expression of (*a*) *HcLyso1* and (*b*) *HcLyso2* was examined in four different tissues. The bacteriolytic activities of *H. cumingii* HcLyso1 protein were measured under different (*c*) temperatures and (*d*) pH. Error bars indicate standard deviation of the mean.

**Figure 4.**
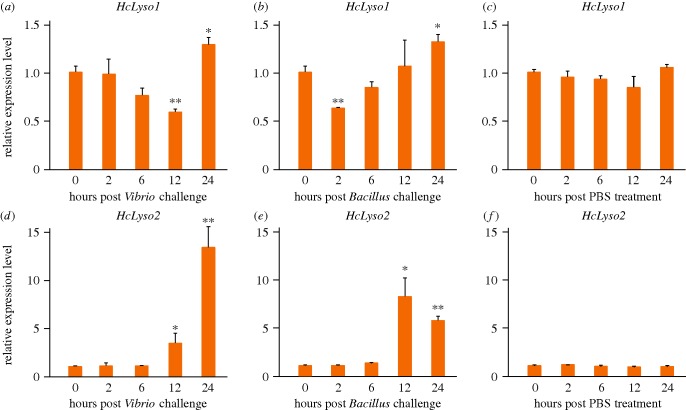
The expression change of the *H. cumingii* BCPL genes after bacterial challenges. (*a,d*) *Vibrio parahemolyticus* and (*b,e*) *B. cereus* were used to challenge *H. cumingii*. (*c,f*) PBS treatment was used as a control. The relative expression levels of (*a–c*) *HcLyso1* and (*d–f*) *Hclyso2* genes were examined in the gills of *H. cumingii*. The difference of gene expression between hour 0 and other time points was analysed using an unpaired sample *t*-test. The significance levels of change were indicated by an asterisk (0.01 < *p* < 0.05) or two asterisks (*p* < 0.01). Error bars indicate standard deviation of the mean.

Because the BCPL proteins are derived from phage-type lysozymes, we hypothesized that the BCPL proteins might possess antibacterial capacity in bivalves. To test this hypothesis, we focused on the *BCPL* genes of *H. cumingii*. The *H. cumingii* genome encodes at least two copies of *BCPL* genes (i.e. *HcLyso1* and *HcLyso2*). We first investigated the expression pattern of the *BCPL* genes of *H. cumingii*. We found evidence that both *HcLyso1* and *HcLyso2* can be expressed in haemocytes, hepatopancreas, gills and mantle (figure 3*a,b*). However, the *HcLyso1* and *HcLyso2* genes have both overlapping and divergent expression patterns: *HcLyso1* has an elevated expression level in hepatopancreas and gills, whereas *HcLyso2* has an elevated expression level in hepatopancreas and mantle. The expression difference indicates a potential functional divergence between *HcLyso1* and *HcLyso2* genes. Nevertheless, these results suggested that both *H. cumingii BCPL* genes are functionally active. Next, we used *Vibrio parahemolyticus* and *Bacillus cereus* to challenge *H. cumingii*. After both bacterial challenges, the *HcLyso1* gene expression level first decreased and then increased, but the *HcLyso2* gene expression level significantly increased (figure 4*a–f*). Finally, we cloned, expressed and purified the HcLyso1 protein and measured its bacteriolytic activity. Our results suggest that the purified HcLyso1 protein exhibits the capacity to degrade bacteria (figure 3*c,d*). The optimal reaction temperature and pH are approximately 30°C and approximately 10.0, respectively (figure 3*c,d*). *Hyriopsis cumingii* is widely distributed within the temperature zone across China. The growth rate is highest from June to October, when the average water temperature is 20–31°C [13]. This fact is compatible with our experimental result, and as bacterial growth rates are typically higher in warmer water, the optimal reaction temperature may reflect this. As for the optimal pH, we suspect it might be related to the specific tissue/cellular environment; however, the possibility that *in vitro* experiments do not reflect the actual lysozyme activity cannot be formally excluded. Taken together, these lines of evidence indicate that the *H. cumingii BCPL* genes respond to bacterial challenges and function in antibacterial activity. It follows that the co-opted lysozymes may augment the immune function of bivalves by actively degrading bacterial cell walls.

## 3. Discussion

HGT, the acquisition of genetic materials from distinct evolutionary lineages, is thought to have played some role in the evolution of eukaryotes. In this study, we report that lysozyme genes previously described in bivalves [9] were horizontally transferred from bacteriophages to two subclasses of bivalves. Subsequently, these horizontally acquired genes underwent both dramatic structural changes in the bivalve genomes—including intron gain and fusion with other genes—as well as recurrent gene duplication. Functional analyses show that these bivalve lysozymes can degrade bacterial cell walls. Therefore, the horizontally acquired lysozyme genes are likely to be acting to augment bivalves’ capacity to restrict bacterial infections. We believe this type of evolutionary strategy for host–microbe interaction might be prevalent over evolutionary time. While phage-type-like lysozyme has been identified in the Manila clam (*R. philippinarum*) [9], that work proposed the occurrence of HGT only as a probable explanation, and did not explore the evolutionary history and mechanism of this lysozyme in details. In this study, we have not only provided evidence that the bivalve genomes acquired phage-type lysozyme genes via HGT, but also explored the detailed evolutionary history and functional consequence of the co-option of a phage lysozyme by bivalves.

Our phylogenetic analyses suggest that the ultimate donors are closely related to the phages isolated in the Mediterranean sea. It seems that the donors and recipients coexisted in the same ecological niche (a water environment), providing physical opportunity for HGT to occur. For the origin of the *BCPL* genes, we propose two competing evolutionary scenarios: (i) direct transfer (an HGT event took place from bacteriophages to bivalves directly); or (ii) transfer via intermediate (the HGT was mediated by bacteria, i.e. bacteriophage to bacteria and then to bivalve). However, the transfer via intermediate scenario requires at least two independent transfer events (bacteriophages to bacteria and bacteria to bivalves), which is less parsimonious than the direct transfer scenario. It is also worth noting that we observed no bacterial sequences within our sequenced genes of interest or the flanking regions we examined, which supports our assertion that the parsimonious direct transfer scenario is the correct one in this case.

We observed that the lysozyme genes from Heterodonta and Palaeoheterodonta form a monophyletic group and the lysozyme gene tree seems largely congruent with the bivalve phylogeny (figure 1). These observations indicate that a single HGT event took place before the divergence of Heterodonta and Palaeoheterodonta. The probability of two or more independent successful transfers from a common source is likely to be low and would be less parsimonious. A recent phylogenomic study reveals the Bivalvia class constitutes a monophyletic group [14]. However, the relationship among the four subclasses of the Bivalvia (Heterodonta, Palaeoheterodonta, Pteriomorphia and Protobranchia) is still controversial [14–17]. If these *BCPL* genes originated from a single HGT event, then it took place before the divergence of Heterodonta and Palaeoheterodonta, but after the divergence of Pteriomorphia and Heteroconchia, given no *BCPL* gene was identified in the complete genomic sequence of *C. gigas* (subclass Pteriomorphia). The origin of Heterodonta is estimated to be approximately 490 million years ago (Ma) [15]. It follows that the most recent common ancestor of Heterodonta and Palaeoheterodonta should be older than 490 Ma, but not older than 520–530 Ma, when Bivalvia arose [15]. This time scale should be taken with caution, as the time scales and the phylogenetic relationships of bivalve species are still controversial [14–17]; however, the conclusion remains that a single event is more likely given our results.

As a potential strategy for dealing with bacteria, exaptation of bactericidal enzymes would represent a potentially fruitful strategy for any eukaryote. Presumably, phage lysozymes have adapted to prey on bacteria long before the evolution of multicellular organisms, and therefore they have been uniquely shaped by evolutionary pressure to affect bacterial cell walls. HGT would allow the eukaryote to ‘short cut’ the slow evolutionary process of adapting an endogenous protein to deal with bacterial infection. However, co-option is of course not without risks to the organism, such as disrupting genomic features and the cellular networks of recipients [18]. While HGT is likely to be random, persistence of horizontally transferred elements would be expected either when they are completely neutral or, as is posited here, when they are adaptive. If they are neutral, degradation is the likely fate of an HGT over a long time scale. This suggests that the persistence of these lysozymes was driven by their utility in the genome, as discussed even through the clear structural shifts and intron acquisition we see.

Recently, bacteriocidal enzymes employed in interbacterial competition, including the type VI secretion amidase effector (Tae) and GH25 lysozyme, have been found to be derived from horizontal transmission from bacteria to eukaryotes or archaea (for GH25 lysozyme). The exapted genes augment the immune function for related eukaryotes or the capacity to compete with bacteria for related archaea [6,19]. In fact, there are many other antagonism genes associated with infection of bacteria by phages or interbacterial competition, which have produced a reservoir for co-option. Therefore, we believe similar co-option might be prevalent within the tree of life, and may be especially prominent among prokaryotes and microbial eukaryotes as HGT has been found to be more frequent in these organisms [1–3,19–21].

## 4. Material and methods

### 4.1. Samples and transcriptome sequencing

The bivalve samples (electronic supplementary material, table S1) were purchased in local markets in Nanjing (Jiangsu Province), Hangzhou (Zhejiang Province) and Wuhu (Anhui Province). The species were identified based on morphological traits and have been confirmed by sequencing mitochondrial *cytochrome c oxidase subunit I* (*cox1*). Throughout this study, RNA was extracted using the RNApure High-purity Total RNA Rapid Extraction kit (spin-column; Bioteke, Beijing, China). The transcriptomes of the 11 bivalve species were sequenced, using an Illumina HiSeqTM 2000. The sequencing reads were de novo assembled, using the Trinity program. We searched for phage-type lysozyme protein homologues (cut-off *E*-value of 10^−05^ with >100 alignable residues), using the tBLASTn algorithm with Enterobacteria phage T4 lysozyme (GenBank accession no. NP_049736.1) as the query.

### 4.2. Cloning the full-length *BCPL* cDNA

To obtain the full-length of the *BCPL* cDNA, we performed both 5′- and 3′-rapid amplification of cDNA ends (RACE), using SMARTer RACE 5′/3′ Kit (Clontech), following the manufacturer's manual. All the primers used in RACE are listed in electronic supplementary material, table S3. The RACE PCR products were sequenced by Springen (Nanjing, China). The full-length *HcLyso2* and *CpLyso1* cDNA sequences were obtained by performing RACE with primers designed based on *CpLyso2* (CpLyso1-F and CpLyso1-R in electronic supplementary material, table S3) and *HcLyso1* (HcLyso2-F and HcLyso2-R in electronic supplementary material, table S3) genes, respectively.

### 4.3. Genome walking in the *BCPL* genes

Genomic DNA was extracted using NucleoSpin Tissue (Clontech). To obtain the flanking genomic regions of the *BCPL* genes, genome walking was carried out using a Universal GenomeWalker 2.0 kit (Clontech) according to the manufacturer's manual. In brief, genomic DNA was digested by *Dra*I, *EcoR*V, *Pvu*II and *Stu*I, separately. The digested genomic DNA was purified using NucleoSpin Gel and PCR Clean-Up kit (Clontech). Each batch of the purified genomic DNA was ligated to the GenomeWalker adaptor. Nested PCRs were then conducted. All the primers used in genome walking were listed in electronic supplementary material, table S4. The resulting PCR amplicons were sequenced by Springen (Nanjing, China). The sequences reported in this paper have been deposited in the GenBank database (accession nos. KT934018-KT934048).

### 4.4. Phylogenetic analysis of lysozymes

The BLASTP algorithm was employed to search against the NCBI non-redundant protein sequence database for the homologues of BCPL proteins. Representative lysozyme sequences were chosen for phylogenetic analysis. Protein sequences were aligned, using the MAFFT multiple sequence alignment program [21] and then manually edited. The phylogenetic tree was inferred with a Bayesian method available in MrBayes v. 3.1.2 [22]. The best-fit substitution model was selected based on ProTest v. 3 [23], and the BLOSUM62 + I + G model was employed. A total of 5 000 000 generations in four chains were run, sampling posterior trees every 100 generations. The first 25% of the posterior trees were discarded. The phylogenetic trees were viewed using FigTree v. 1.4.2.

### 4.5. Expression of fusion genes

The expression of fusion genes, *PGRP-PaLyso1* of *P. abrupta*, *MLRD2-MiLyso1* of *M. iridescens*,and *HSPG-WbLyso2* of Wenbei, were carried out using 5′-RACE cDNA with the mixture of UPM-long and UPM-short primers and lysozyme gene-specific primers listed in electronic supplementary material, table S5.

### 4.6. Expression pattern of *HcLyso1* and *HcLyso2*

Haemocytes, hepatopancreas, gills and mantle were sampled from healthy *H. cumingii* for RNA isolation. Diluted *V. Parahemolyticus* and *B. cereus* cultures (approx. 3 × 10^7^ cells) were injected into the adductor muscles of *H. cumingii*. As a control, *H. cumingii* was also treated with the phosphate-buffered saline (PBS) solution. At 0, 2, 6, 12 and 24 h post-bacterial challenge or PBS treatment, the gills from three *H. cumingii* were sampled for RNA extraction. RNA was reverse transcribed with PrimeScript RT Master Mix (Perfect Real Time) for qRT-PCR analysis. For qRT-PCR analysis, we used SYBR Premix Ex Taq II (Tli RNaseH Plus; Dalian, China) and the *actin* gene as a reference. All the samples were repeated in triplicate. The 2^−ΔΔCt^ method was used to analyse the relative changes in gene expression [24]. The difference of gene expression between hour 0 and other time points was analysed using an unpaired-sample *t*-test.

### 4.7. Bacteriolytic activity of *HcLyso1* protein

In-Fusion PCR Cloning kit (Clontech) was used for the construction of recombinant vector. The *HcLyso1* gene (the whole open reading frame, 456 bp) was obtained using gene-specific primers with 16 bp vector sequences at the 5′ end and Clontech SeqAmp DNA polymerase. The amplified gene fragment was then cloned into the pET30a vector linearized by *Eco*RI and *Xho*I. The recombinant vector was transformed into Trans-T1 cells, and the positive clone was sequenced to ensure correct insertion. The recombinant vector was then transformed into BL21(DE3) cells for protein expression. After denaturation and renaturation as described previously [25], the recombinant protein with His-tag was purified using His·Bind resin chromatography (Novagen).

The bacteriolytic activity of HcLyso1 at different temperature or pH levels was analysed using lysozyme assay kit (Jiancheng, Nanjing, China). In principle, lysozyme activity is measured by tracking the increase in transmittance as the enzyme degrades the bacterial cell wall. A total of three mixtures were prepared: blank mixture (0.2 ml distilled water, 2.0 ml working solution *Micrococcus luteus*), standard mixture (0.2 ml of standard lysozyme solution, 2.0 ml working solution *M. luteus*) and sample mixture (0.2 ml HcLyso1 solution, 2.0 ml working solution *M. luteus*). The bacteriolytic activity of HcLyso1 can be calculated through the following formula:
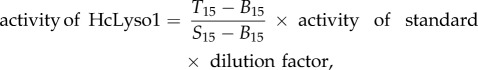
where *T*_15_ represents the transmittance at 530 nm after the sample mixture was treated under different temperature or pH levels for 15 min; *B*_15_ represents the transmittance at 530 nm after the blank mixture was placed in 37°C for 15 min; *S*_15_ represents the transmittance at 530 nm after standard mixture was placed in 37°C for 15 min; and the activity of standard is 200 U ml^−1^.

## Supplementary Material

Supplementary material in support of Bacteriophage Co-option

## Data accessibility

The sequences reported in this paper have been deposited in the GenBank database (accession nos KT934018-KT934048). Any constructs created are available upon request. The author responsible for distribution of materials integral to the findings presented in this article is G.-Z.H. (guanzhu@njnu.edu.cn).

## References

[1] Koonin EV, Makarova KS, Aravind L 2001 Horizontal gene transfer in prokaryotes: quantification and classification. Annu. Rev. Microbiol. 55, 709–742. (doi:10.1146/annurev.micro.55.1.709) 11544372 10.1146/annurev.micro.55.1.709 PMC4781227

[2] Gogarten JP, Townsend JP 2005 Horizontal gene transfer, genome innovation and evolution. Nat. Rev. Microbiol. 3, 679–687. (doi:10.1038/nrmicro1204) 16138096 10.1038/nrmicro1204

[3] Keeling PJ, Palmer JD 2008 Horizontal gene transfer in eukaryotic evolution. Nat. Rev. Genet. 9, 605–618. (doi:10.1038/nrg2386) 18591983 10.1038/nrg2386

[4] Monzingo AF, Marcotte EM, Hart PJ, Robertus JD 1996 Chitinases, chitosanases, and lysozymes can be divided into procaryotic and eucaryotic families sharing a conserved core. Nat. Struct. Biol. 3, 133–140. (doi:10.1038/nsb0296-133) 8564539 10.1038/nsb0296-133

[5] Bachali S, Jager M, Hassanin A, Schoentgen F, Jollès P, Fiala-Medioni A 2002 Phylogenetic analysis of invertebrate lysozymes and the evolution of lysozyme function. J. Mol. Evol. 54, 652–664. (doi:10.1007/s00239-001-0061-6) 11965437 10.1007/s00239-001-0061-6

[6] Schulenburg H, Boehnisch C 2008 Diversification and adaptive sequence evolution of *Caenorhabditis* lysozymes (Nematoda: Rhabditidae). BMC Evol. Biol. 8, 114 (doi:10.1186/1471-2148-8-114) 18423043 10.1186/1471-2148-8-114 PMC2383907

[7] Callewaert L, Michiels CW 2010 Lysozymes in the animal kingdom. J. Biosci. 35, 127–160. (doi:10.1007/s12038-010-0015-5) 20413917 10.1007/s12038-010-0015-5

[8] Callewaert L, Van Herreweghe JM, Vanderkelen L, Leysen S, Voet A, Michiels CW 2012 Guards of the great wall: bacterial lysozyme inhibitors. Trends Microbiol. 20, 501–510. (doi:10.1016/j.tim.2012.06.005) 22840966 10.1016/j.tim.2012.06.005

[9] Ding J, Wang R, Yang F, Zhao L, Qin Y, Zhang G, Yan X 2014 Identification and characterization of a novel phage-type like lysozyme from Manila clam, *Ruditapes philippinarum*. Dev. Comp. Immunol. 47, 81–89. (doi:10.1016/j.dci.2014.06.013) 24995730 10.1016/j.dci.2014.06.013

[10] Metcalf JA, Funkhouser-Jones LJ, Brileya K, Reysenbach AL, Bordenstein SR 2014 Antibacterial gene transfer across the tree of life. Elife 3, e04266 (doi:10.7554/eLife.04266) 10.7554/eLife.04266 PMC4241558 25422936

[11] Mizuno CM, Rodriguez-Valera F, Kimes NE, Ghai R 2013 Expanding the marine virosphere using metagenomics. PLoS Genet. 9, e1003987 (doi:10.1371/journal.pgen.1003987) 24348267 10.1371/journal.pgen.1003987 PMC3861242

[12] Ghai R et al. 2010 Metagenome of the Mediterranean deep chlorophyll maximum studied by direct and fosmid library 454 pyrosequencing. ISME J. 4, 1154–1166. (doi:10.1038/ismej.2010.44) 20393571 10.1038/ismej.2010.44

[13] Xu Z, Xu Z, Wu Q 1988 Effects of temperature on the physiology of *Hyriopsis cumingii*. Fish Sci. 7, 9–11.

[14] Kocot KM et al. 2011 Phylogenomics reveals deep molluscan relationships. Nature 477, 452–456. (doi:10.1038/nature10382) 21892190 10.1038/nature10382 PMC4024475

[15] Plazzi F, Passamonti M 2010 Towards a molecular phylogeny of Mollusks: bivalves’ early evolution as revealed by mitochondrial genes. Mol. Phylogenet. Evol. 57, 641–657. (doi:10.1016/j.ympev.2010.08.032) 20817110 10.1016/j.ympev.2010.08.032

[16] González VL, Andrade SC, Bieler R, Collins TM, Dunn CW, Mikkelsen PM, Taylor JD, Giribet G 2015 A phylogenetic backbone for Bivalvia: an RNA-seq approach. Proc. R. Soc. B 282, 20142332 (doi:10.1098/rspb.2014.2332) 10.1098/rspb.2014.2332 PMC4308999 25589608

[17] Roy K, Hunt G, Jablonski D 2009 Phylogenetic conservatism of extinctions in marine bivalves. Science 325, 733–737. (doi:10.1126/science.1173073) 19661426 10.1126/science.1173073

[18] Baltrus DA 2013 Exploring the costs of horizontal gene transfer. Trends Ecol. Evol. 28, 489–495. (doi:10.1016/j.tree.2013.04.002) 23706556 10.1016/j.tree.2013.04.002

[19] Nelson-Sathi S et al. 2015 Origins of major archaeal clades correspond to gene acquisitions from bacteria. Nature 517, 77–80. (doi:10.1038/nature13805) 25317564 10.1038/nature13805 PMC4285555

[20] Andersson JO 2009 Gene transfer and diversification of microbial eukaryotes. Annu. Rev. Microbiol. 63, 177–193. (doi:10.1146/annurev.micro.091208.073203) 19575565 10.1146/annurev.micro.091208.073203

[21] Katoh K, Standley DM 2013 MAFFT multiple sequence alignment software version 7: improvements in performance and usability. Mol. Biol. Evol. 30, 772–780. (doi:10.1093/molbev/mst010) 23329690 10.1093/molbev/mst010 PMC3603318

[22] Ronquist F, Huelsenbeck JP 2003 MrBayes 3: Bayesian phylogenetic inference under mixed models. Bioinformatics 19, 1572–1574. (doi:10.1093/bioinformatics/btg180) 12912839 10.1093/bioinformatics/btg180

[23] Darriba D, Taboada GL, Doallo R, Posada D 2011 ProtTest 3: fast selection of best-fit models of protein evolution. Bioinformatics 27, 1164–1165. (doi:10.1093/bioinformatics/btr088) 21335321 10.1093/bioinformatics/btr088 PMC5215816

[24] Livak KJ, Schmittgen TD 2001 Analysis of relative gene expression data using real-time quantitative PCR and the 2^-ΔΔ*C*_T_^ method. Methods 25, 402–408. (doi:10.1006/meth.2001.1262) 11846609 10.1006/meth.2001.1262

[25] Lan ZF, Zhou J, Zhang XW, Wang ZH, Zhao XF, Ren Q, Wang J-X 2013 Characterization of an immune deficiency homolog (IMD) in shrimp (*Fenneropenaeus chinensis*) and crayfish (*Procambarus clarkii*). Dev. Comp. Immunol. 41, 608–617. (doi:10.1016/j.dci.2013.07.004) 23850721 10.1016/j.dci.2013.07.004

