# Supplementary material for: Co-option of bacteriophage lysozyme genes by bivalve genomes
Source: Open Biol. 2017 Jan 18;7(1):160285. doi: 10.1098/rsob.160285 (PMC5303276; doi:10.1098/rsob.160285)
Supplement: Supplementary material in support of Bacteriophage Co-option [file rsob160285supp1.pdf]

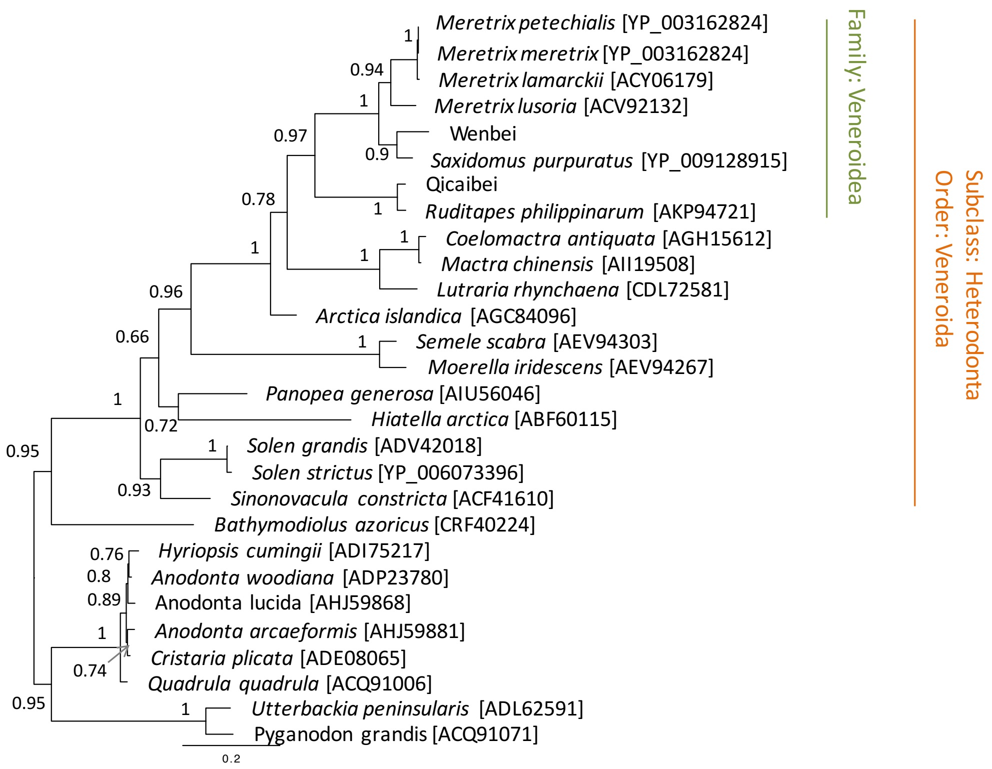

Supplementary Fig. 1. Phylogenetic relationship of bivalve species. The phylogenetic tree is reconstructed based on mitochondrial *cytochrome c oxidase subunit I* (*cox1*) protein sequences. The topology is inferred using an approximately maximum-likelihood method. The numbers on the selected nodes represent SH-like local support value.

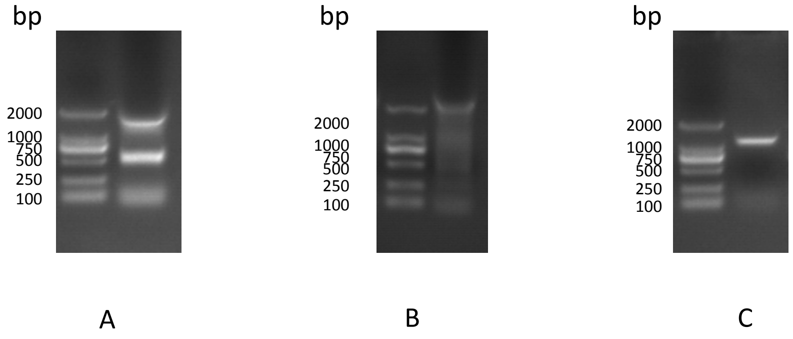

Supplementary Fig. 2. Expression of fusion genes: *PGRP-PaLyso1* of *P. abrupta* (**A**), *MLRD2-MiLyso1* of *M. iridescens* (**B**), and *HSPG-WbLyso2* of Wenbei (**C**). These analyses were carried out using 5'-RACE cDNA with the mixture of UPM-long and UMP-short primers and lysozyme gene-specific primers (Table S5). The longer band indicates the expression of the fusion gene, and the shorter band indicates the expression of lysozyme gene.

Table S1. Species used in this study

Species	Subclass	Order	Family
Saxidomrs purpuratus	Heterodonta	Veneroida	Veneridae
Meretrix meretrix	Heterodonta	Veneroida	Veneridae
Ruditapes philippinarum	Heterodonta	Veneroida	Veneridae
Moerella iridescens	Heterodonta	Veneroida	Tellinidae
Panopea abrupta	Heterodonta	Myoida	Hiatellidae
Solen strictus	Heterodonta	Veneroida	Solenidae
Wenbei[1]	Heterodonta	Veneroida	Veneridae
Qicaibei[1]	Heterodonta	Veneroida	Veneridae
Hyriopsis cumingii	Palaeoheterodonta	Unionoida	Unionidae
Cristaria plicata	Palaeoheterodonta	Unionoida	Unionidae
Anodonta woodiana	Palaeoheterodonta	Unionoida	Unionidae

[1]Latin name currently unavailable

Supplementary Table 2. Summary of bivalve lysozyme gene information

Contig No.	Contig length	E-value	Identities	Gene Name	Accession No.
**Wenbei**					
Unigene11242	1486	5.00E-09	43/149 (29%)	WbLyso1	KT934022
Unigene68894*	180			WbLyso2	KT934045/KT934043
Solen strictus					
Unigene5822	622	1.00E-13	42/152 (28%)	SsLyso2	KT934020/KT934033
Unigene52591	919	1.00E-10	43/144 (30%)	SsLyso1	KT934021/KT934034
Saxidomus purpuratus					
Unigene35766	632	4.00E-11	44/149 (30%)	SpLyso1	KT934023/KT934035
Ruditapes philippinarum					
Unigene25660	674	3.00E-16	44/148 (30%)	RpLyso2	KT934027/KT934038
Unigene32476	820	2.00E-12	43/148 (29%)	RpLyso1	KT934026
**Qicaibei**					
Unigene4847	1435	2.00E-14	43/148 (29%)	QbLyso2	KT934025/KT934037
Unigene17195	976	5.00E-12	41/149 (28%)	QbLyso1	KT934024/KT934036
Panopea abrupta					
CL5507	2260	4.00E-13	43/150 (29%)	PaLyso1	KT934028/KT934039
Moerella iridescens					
CL3011	1586	4.00E-14	45/145 (31%)	MiLyso1	KT934046
Meretrix meretrix					
CL6411	651	1.00E-13	45/151 (30%)	MmLyso2	KT934019
Unigene24890	1150	1.00E-10	45/157 (29%)	MmLyso1	KT934018
Hyriopsis cumingii					
comp36715	513	4.00E-19	47/148 (32%)	HcLyso1<br>HcLyso2	KT934031/KT934042<br>KT934032
Cristaria plicata					
Unigene17248	869	3.00E-20	51/164 (31%)	CpLyso2	KT934030
CL7588	687	6.00E-16	45/144 (31%)	CpLyso3<br>CpLyso1	KT934044<br>KT934029
Anodonta woodiana					
Unigene73183	441	4.00E-17	43/135 (32%)	AwLyso1	KT934041

Note: * This hit (E-value of e-16, identities of 57% [34/60], and spanning the whole contig) was obtained by employing the tBLASTn algorithm against the Wenbei transcriptome sequence with WbLyso1 as the query.

Supplementary Table 3. Primers used in RACE

AwLyso-F: 5'-GGAGGCTCAGCTCGTTATCGCCAATAT-3'
AwLyso-R: 5'-TGTCCACCATCTCGTCTGCTGCTTTAG-3'
SsLyso1-F: 5'-GGCGACACTGACAGAGCAGGAAATGAC-3'
SsLyso2-F: 5'-AACTTGGGAATCAGCAGAATGAACGGC-3'
MmLyso1-F: 5'-TAAGAGCAGAGGAGAAAGGTTGGTGGC-3'
MmLyso1-R: 5'-GCCACCAACCTTTCTCCTCTGCTCTTA-3'
MmLyso2-F: 5'-GCTACCTTACATTCGGCATTGGGCATC-3'
MmLyso2-R: 5'-AGCAGTCGTGGAAGATGGAATGAGTCG-3'
SpLyso-F: 5'-GAAAAGCAGGGCAGTGCGATTAGTGGC-3'
SpLyso-R: 5'-ATTCTTGCCACTAATCGCACTGCCCTG-3'
QcbLyso1-F: 5'-GCATTCAACCTTGGGGGACGGCTTAGAG-3'
QcbLyso1-R: 5'-TTCATTTCATCAGCAGCCGTATTCCAGT-3'
QcbLyso2-F: 5'-CGTGCGTCGTCCTTGTTGTCGTAGTGT-3'
QcbLyso2-R: 5'-TCGGGGTCGTTATGTGTGACAAGATGG-3'
RpLyso1-F: 5'-TACCCTGAATGCGAGACGTGGCCTAGTG-3'
RpLyso1-R: 5'-CACTAGGCCACGTCTCGCATTCAGGGTA-3'
RpLyso2-F: 5'-TTGGACATCTCATCACTCACAACGACCC-3'
RpLyso2-R: 5'-CTCGGGGTCGTTGTGAGTGATGAGATGT-3'
MiLyso-F: 5'-TGATTGCCAAGATGGGAAGAGGCTACC-3'
PaLyso-F: 5'-CGACTCGTGGAGAGGATGCGAAATGTG-3'
PaLyso-R: 5'-TTTCGCATCCTCTCCACGAGTCGCACC-3'
WbLyso1-F: 5'-GAAAAGCAGAGCAGTGCGATTAGTGGC-3'
WbLyso1-R: 5'-GCCACTAATCGCACTGCTCTGCTTTTC-3'
WbLyso2-F: 5'-GGTGACAAAACTATTCCTGCCTCGTGG-3'
WbLyso2-R: 5'-GCACTAACGCTTGTTCCTACTGGTTTCCC-3'
CpLyso1-F: 5'-CCAGAATACGGCAAGTCAGTTGGCAGC-3'
CpLyso1-R: 5'-GGAAGTCTGGGAAAAGTCGGGAGCATC-3'
CpLyso2-F: 5'-CCACGAGTGACCCAGAATATGGCAAGC-3'
CpLyso2-R: 5'-CTTGCTGACAGGAGTACCTAATCGCTTGCC-3'
CpLyso3-F: 5'-CCAAAATCTTCCGAATGAGGTCCAGC-3'
CpLyso3-R: 5'-CCGTTTGGCTCTATTGGGCACTTGTCC-3'
HcLyso1-F: 5'-TCCAGAATACGGCAAGTCAGTTGGCAGC-3'
HcLyso1-R: 5'-GGGCTGCCAACTGACTTGCCGTATTCT-3'
HcLyso2-F: 5'-CCAGAAGAGGCTATGCTCGTCATCGTT-3'
HcLyso2-R: 5'-GTCTGCGGCTTTGGACCAGTCTTGTG-3'

UPM-Long:
CTAATACGACTCACTATAGGGCAAGCAGTGGTATCAACGCAGAGT
UPM-Short: CTAATACGACTCACTATAGGGC
5'-CDS Primer A T_{25}VN
SMARTer ⅡA oligo AAGCAGTGGTATCAACGCAGAGTACXXXXX
3'-CDS primer AAAGCAGTGGTATCAACGCAGAGTAC(T)30VN

Note:
X= undisclosed base in the proprietary SMARTer oligo sequence
N = A, C, G, or T;
V= A, G, or C

Supplementary Table 4. Primers used in genome walking

SpLyso-walk-R1: 5’-GCCACTAATCGCACTGCCCTGCTTTTC-3’
SpLyso-walk-R2: 5’-CATCAGCAGCCTTATTCCAGTCCTTGT-3’
RpLyso1-walk-R1: 5’-CCACGTCTCGCATTCAGGGTAGATTTC-3’
RpLyso1-walk-R2: 5’-CCTCATCTACCTTCAGCTGGGCTTTGA-3’
RpLyso1-walk-F1: 5’-GAGCGGTTACATCTCGCTGCATAGTGC-3’
RpLyso1-walk-F2: 5’-GGAACTGCGTCAATCCCTTGATGACTT-3’
RpLyso1-walk-F3: 5’-CGCCAAGAAACTGCTTGTCATGCCTTTG-3’
RpLyso1-walk-F4: 5’-GGTTACCAGTCGATACTGCCTCGTTTCC-3’
RpLyso2-walk-R1: 5’-CAGTGTGGGAAGATGGAATGGAAGGTA-3’
RpLyso2-walk-R2: 5’-CGTGTGTTCCCGCTGGTTTCCCGTACT-3’
SsLyso1-walk-R1: 5’-AGCGTTTGAGTCACTTATGGCTGTGTC-3’
SsLyso1-walk-R2: 5’-CCCTCGCTGTCCTTGTAGATACTCTCA-3’
SsLyso1-walk-F1: 5’-ATGGCGACACTGACAGAGCAGGAAATG-3’
SsLyso1-walk-F2: 5’-AAGGCACAGCTGGTTATCGACGAAGGT-3’
SsLyso1-gF: 5’-TGCAAGCCTCCAATGACTTGGAGTGAG-3’
SsLyso1-gR: 5’-CAACTTGCCCGTACCACTTGCTGTTCT-3’
SsLyso1-walk-R3: 5’-ACTCCGGTCTGGCAGTTGACAATATCC-3’
SsLyso1-walk-R4: 5’-CTGCCACCAATTTTTGCAACGTGTACG-3’
SsLyso1-walk-R5: 5’-CCGTCGGATGCAATGTTCAGAATGTGT-3’
SsLyso1-walk-R6: 5’-ACCCGAAGACTGTTCCTTTCCCGAATG-3’
SsLyso2-walk-R1: 5’-GGTTCAATCTCAGCTCGGAACGGTATG-3’
SsLyso2-walk-R2: 5’-ACTCCAACCTTTACCAACGGCGTAACA-3’
SsLyso2-walk-F1: 5’-AGAGCAGCAGACGAAATGGAAAACAGC-3’
SsLyso2-walk-F2: 5’-GGGCAGAAAGAATGTTACGCCGTTGGT-3’
SsLyso2-gF: 5’-ACATGGACCCACTCGGTAATCCAACGT-3’
SsLyso2-gR: 5’-ATACCAGCGGTGGCCAGGTTACGTTAC-3’
PaLyso-gF: 5’-TGGTCTTTTGCAGTTCGAATTCATCGC-3’
PaLyso-gR: 5’-TCTTGAACTTCCTTAGGTTTCCTCCCA-3’
PaLyso-walk-R1: 5’-CACCGGCCCTTTTTTATGCGGATGTTA-3’
PaLyso-walk-R2: 5’-GCGGTAACTTCCTCCAAACGTTGCGAT-3’
PaLyso-walk-R3: 5’-TATCAGTGGCGTCGGCAAGGAGGTAGTC-3’
PaLyso-walk-R4: 5’-TGTCAAGGATCCCGTGCTGTCTGAACT-3’
PaLyso-walk-R5: 5’-TTTGGCATTTGGAAGCAGACTTGTGAA-3’
PaLyso-walk-R6: 5’-CAATGAAAGCCGCCGCAAATGAAACTC-3’
MiLyso-walk-F1: 5’-GCTGCCCTTGACGACAGGAACTGGAAC-3’
MiLyso-walk-F2: 5’-AACTGGAACAAGGCGGCGGATGAGATG-3’
MiLyso-walk-R1: 5’-CGAGCCCGTTTGAGGTGAGCCAGATTT-3’
MiLyso-walk-R2: 5’-CCATCTCATCCGCCGCCTTGTTCCAGT-3’
MiLyso-walk-R3: 5’-CCTCTTCCCATCTTGGCAATCAACTCA-3’
MiLyso-walk-R4: 5’-TGCCTGACAGTGTATTCCAGACAACGC-3’
HcLyso1-walk-F1: 5’-ACACAGACCACCTGGGCTATTTGCACT-3’
HcLyso1-walk-F2: 5’-CCTGGGCTATTTGCACTTTGGTATTGG-3’
HcLyso1-walk-F3: 5’-AATCTGGGAGAGACCAAACTCGCAAAG-3’

HcLyso1-walk-F4: 5’-GCGGGTGGAGTTCGTAGCACTATCGTC-3’
HcLyso1-walk-R1: 5’-CGGGCTGCCAACTGACTTGCCGTATTC-3’
HcLyso1-walk-R2: 5’-CGGTGATAAGGTGACCAATACCAAAGT-3’
HcLyso1-walk-R3: 5’-ATGGTCGGAGTGAAGTCTGCCAGCTCA-3’
HcLyso1-walk-R4: 5’-CTTGGAGCAGACCCTACGGAGCCAGTT-3’
HcLyso2-walk-R1: 5’-CGTCTGCGGCTTTGGACCAGTCTTGTG-3’
HcLyso2-walk-R2: 5’-TGAAAAGCCTGGAGCATCCCGCTAAAG-3’
HcLyso2-walk-F1: 5’-CCACCCGACTTTTGGCATAGGTCATCT-3’
HcLyso2-walk-F2: 5’-GTGACCCAGAAAACGGCAAGCCAGTAG-3’
CpLyso1-walk-F1: 5’-TTGGTATTGGTCACCTTATCACCGAAA-3’
CpLyso1-walk-F2: 5’-TGCTCCCGACTTTTCCCAGACTTCCAG-3’
CpLyso1-walk-R1: 5’-ACCTGCTGTCCACCATCTCGTCTGCT-3’
CpLyso1-walk-R2: 5’-GAAGTCTGGGAAAAGTCGGGAGCATC-3’
CpLyso1-walk-R3: 5’-GGGGGAGGGAAACACCCAACAGTTCCT-3’
CpLyso1-walk-R4: 5’-CCCTCGCCTATTTGATCTTTGCATTCG-3’
CpLyso1-walk-R5: 5’-GCCGTCCGTCAACGTGGTGAGATTTAC-3’
CpLyso1-walk-R6: 5’-CCCCTGACTTACGCAATCGCAGACAGA-3’
CpLyso2-walk-F1: 5’-CGAGGAAGTTTTGGGAAAGACATTGA-3’
CpLyso2-walk-F2: 5’-CTTTGGCGGGATGCTCAAGGCTTTTTA-3’
CpLyso2-walk-F3: 5’-CGGCGGCAGTTGGTGTCAATCCGAAAT-3’
CpLyso2-walk-F4: 5’-TGGCAACTTCACTGCATGACAATAGGG-3’
CpLyso2-walk-F5: 5’-CCTGATTGCATTGTGCCGTCGTAAATT-3’
CpLyso2-walk-F6: 5’-TGCCGCAACACTCGAGCGGAGATATAC-3’
CpLyso2-walk-F7: 5’-TAACAACGGCGAGCAGCAAAAACACCA-3’
CpLyso2-walk-F8: 5’-GCGGGCTCTCCACGGGGTAATCTAAAC-3’
CpLyso2-walk-F9: 5’-ACGAGATGATTTCGCTGTTGGAAGTGA-3’
CpLyso2-walk-F10: 5’-CAACGAGGTGCAACTGCGATGAGACAT-3’
CpLyso2-walk-R1: 5’-CCACATGCTGTTTTCCATTTCGATTGC-3’
CpLyso2-walk-R2: 5’-CGATGACGAGCATAGCCTCTTCTGGAA-3’
CpLyso2-walk-R3: 5’-CGATGACGAGCATAGCCTCTTCTGGAA-3’
CpLyso2-walk-R4: 5’-CCAAAAGTCAGGTTGCCAAGGTGATCC-3’
CpLyso2-walk-R5: 5’-CAATATCCACCCCACCCACCCCCTACT-3’
CpLyso2-walk-R6: 5’-CACCCCCTACTGATGGGCTGGGAGTGA-3’
WbLyso1-walk-R1: 5’-GCCACTAATCGCACTGCTCTGCTTTTC-3’
WbLyso1-walk-R2: 5’-TCAGTTTTCCTCCAAGGTTGAATGCCA-3’
WbLyso1-walk-R3: 5’-GGCCGTAGGCAGGTATTGTTTTCACGT-3’
WbLyso1-walk-R4: 5’-CAACAATCACAACCGAGGCAGCGAGAT-3’
WbLyso1-walk-R5: 5’-TGTTCACATTATGCCCTGAGGGTCCAA-3’
WbLyso1-walk-R6: 5’-TTTACCACGCCCATGGTTCCTATTGTG-3’
WbLyso1-walk-F1: 5’-AAAGGAAGACGAGGGTTTTGTTGCCAA-3’
WbLyso1-walk-F2: 5’-ACATGGCCTGGCGAAGTGAAAGAGATC-3’
QcbLyso1-gF: 5’-CTGTTCTCGTCATGTGACCAAACTGAAT-3’
QcbLyso1-gR: 5’-GCACGAAGAAAATGGTGATGACGAACA-3’
QcbLyso1-walk-R1: 5’-TTCAGTTTGGTCACATGACGAGAACAG-3’
QcbLyso1-walk-R2: 5’-TTGGTCACATGACGAGAACAGAACAGA-3’

QcbLyso2-walk-R1: 5’-GGAATGGAAGGTGTTTACTGCCGATGT-3’ QcbLyso2-walk-R2: 5’-TCGGGGTCGTTATGTGTGACAAGATGG-3’
QcbLyso2-walk-R3: 5’-GTGCCTTGAATACGCATGTTTTCCCTA-3’
QcbLyso2-walk-R4: 5’-GTTCACAGTTTTTCGGACAAGCACGTT-3’
QcbLyso2-walk-R5: 5’-GAATGCGCTACCGCTCGGTCATAATGG-3’
QcbLyso2-walk-R6: 5’-CGTGTCAGGCATACATGACAAAGGTCA-3’
AP1: 5’-GTAATACGACTCACTATAGGGC-3’
AP2: 5’-ACTATAGGGCACGCGTGGT-3’

Supplementary Table 5. Primers used in gene expression analyses

Tissue-specific expression
HcLyso1-ex-F: 5’-TATCGGATCCGAATTCATGGCTGCATCTTCAAACTC-3’
HcLyso1-ex-R: 5’-GGTGGTGGTGCTCGAGATCAGCCAAGTTTCTTATTC-3’

qRT-PCR
HcLyso1-RT-F: 5’-TGCAAGGAGAGAGTGGATGAA-3’
HcLyso1-RT-R: 5’-TATTGGCGATAACGAGCTGAG-3’
HcLyso2-RT-F: 5’-CACCAAGTGTGACCCAGAAA-3’
HcLyso2-RT-R: 5’-TAACGATGACGAGCATAGCC-3’
Hc-Actin-RT-F: 5’-GTGGCTACTCCTTCACAACC-3’
Hc-Actin-RT-R: 5’-GAAGCTAGGCTGGAACAAGG-3’

Expression of fusion genes UPM-Long:
CTAATACGACTCACTATAGGGCAAGCAGTGGTATCAACGCAGAGT
UPM-Short: CTAATACGACTCACTATAGGGC

WbLyso2-RACE-R2: 5’-CCCTGTCCCAGTCCCTTTCCCCCATTG-3’
MiLyso1-RACE-R: 5’-ATCTCATCCGCCGCCTTGTTCCAGTTCC-3’
PaLyso1-RACE-R3: 5’-TTAGGTTTCCTCCCAGGTTGAATGCCA-3’
